# Shikonin ameliorated mice colitis by inhibiting dimerization and tetramerization of PKM2 in macrophages

**DOI:** 10.3389/fphar.2022.926945

**Published:** 2022-08-17

**Authors:** Baoyuan Huang, Qiumei Wang, Lin Jiang, Shuru Lu, Chengcheng Li, Chunqi Xu, Caiyan Wang, Enxin Zhang, Xiaojun Zhang

**Affiliations:** ^1^ School of Pharmaceutical Science, Guangzhou University of Chinese Medicine, Guangzhou, China; ^2^ International Institute of Translational Chinese Medicine, Guangzhou University of Chinese Medicine, Guangzhou, China; ^3^ Shenzhen Hospital of Guangzhou University of Chinese Medicine, Shenzhen, China

**Keywords:** shikonin, PKM2, polymer, ulcerative colitis, macrophage

## Abstract

Dysregulated immune response plays a pivotal role in Ulcerative colitis. In lamina propria of inflammatory colonic mucosa, macrophages tend to polarize into M1 type and metabolically reprogram to aerobic glycolysis. PKM2 orchestrates glucose metabolic switch in macrophages, which tetramer has high pyruvate kinase activity, while which dimer mainly works as a protein kinase to stabilize HIF-1α and mediate anabolism. Shikonin is a potent PKM2 inhibitor derived from traditional Chinese medicine Arnebiae Radix with anti-inflammatory and anticarcinogen activities. However, it is unclear which conformation of PKM2 is inhibited by Shikonin, and whether this inhibition mediates pharmacological effect of Shikonin. In this study, we examined the efficacy of Shikonin on dextran sulfate sodium-induced mice colitis and determined the states of PKM2 aggregation after Shikonin treatment. Results showed that Shikonin dose-dependently alleviated mice colitis, down-regulated expression of F4/80, iNOS and CD86, decreased IFN-γ, IL-1β, IL-6 and TNF-α, while increased IL-10 in mice colon. Furthermore, Shikonin suppressed the pyruvate, lactate production and glucose consumption, inhibited the pyruvate kinase activity and nuclear translocation of PKM2, and decreased both dimerization and tetramerization of PKM2 in macrophages. *In vitro* assay revealed that Shikonin bounded to PKM2 protein, inhibited the formation of both dimer and tetramer, while promoted aggregation of PKM2 macromolecular polymer. TEPP-46, an activator of PKM2 tetramerization, attenuated the ameliorative effect of Shikonin on disuccinimidyl suberate mice. In summary, Shikonin improved mice colitis, which mechanism may be mediated by inhibiting dimerization and tetramerization of PKM2, suppressing aerobic glycolysis reprogram, improving mitochondrial dynamic, and therefore alleviating inflammatory response of macrophages.

## Introduction

Ulcerative colitis (UC) is a complex chronic inflammatory disease which begins with inflammation of the rectal mucosa and extends proximally, affecting any aspect of colon (Kobayashi et al., 2020). Typical symptoms include relapsed abdominal pain, diarrhea, and hematochezia (Kucharzik et al., 2020). The high recurrence rate and undermined quality of life in UC patients place increasing burdens on the healthcare system through direct or indirect costs (Ananthakrishnan, 2015). Although medications involving 5-aminosalicylic acid, corticosteroids and immunosuppressants can relieve symptoms of UC, their clinical applications are limited by inevitable side effects, such as nausea, myelosuppression and risk of carcinogenesis (Ungaro et al., 2017; Neurath and Leppkes, 2019). The etiology and pathogenesis of UC are generally believed to include multiple contributors: genetic susceptibility, immune response, microbial dysbiosis, depression, etc. In which, the immune response is a complex physiological process and plays a crucial role (Ray, 2017; Tatiya-aphiradee et al., 2018; Neurath, 2019).

Macrophages are essential components of innate immunity and key regulators of intestinal microenvironment homeostasis (Na et al., 2019). In lamina propria of inflammatory colon, plenty of macrophages polarize into M1 type and release a large number of pro-inflammatory cytokines, which mitochondrial dynamic balance is impaired, accompanied by a metabolic reprogramming from oxidative phosphorylation to aerobic glycolysis (Breda et al., 2019; Viola et al., 2019; Wang et al., 2021). PKM2 is a key metabolic enzyme, which dimer and tetramer, although composed of the same monomer, play distinctly different roles. PKM2 tetramers, expressed mainly in the cytoplasm of proliferating cells, has high pyruvate kinase activity (Zhang et al., 2019; Liu et al., 2021). However, phosphorylation of PKM2 by tyrosine 105 residue (Tyr-105) inhibits the tetramer activity and stabilizes the dimer structure, resulting in translocation to nucleus, where the dimer works mainly as protein kinase to active HIF-1α and mediate aerobic glycolytic metabolic reprogramming in pro-inflammatory macrophages and cancer cells (Hitosugi et al., 2009; Palsson-McDermott et al., 2015; Shirai et al., 2016).

Shikonin, a natural naphthoquinone compound, is the main active ingredient derived from traditional Chinese medicine Arnebiae Radix, which is the dried root of *Arnebia euchroma* (Royle) Johnst or *Arnebia guttata* Bunge (Guo et al., 2019). It has anti-inflammatory, antibacterial, anti-tumor and hypoglycemic activities (Sun et al., 2022). Previous data have shown that Shikonin is an inhibitor of PKM2 and can relieve DSS-induced mice colitis (Chen et al., 2011; Andujar et al., 2012; Andujar et al., 2013; Han et al., 2021). However, as is known that PKM2 tetramer and dimer execute desperate metabolic functions, which may skew macrophages to anti-inflammation and pro-inflammation respectively, it should be clarified that which specific formation of PKM2 inhibited by Shikonin, will subsequently alter immunophenotypes of macrophages, and whether this inhibition is related to anti-inflammatory effect of Shikonin.

In this study, we firstly assessed the protective effect of Shikonin on DSS-induced mice colitis and examined its regulation on macrophage infiltration and polarization in DSS mice colon. Subsequently, the inhibitory effect of Shikonin on PKM2 activity and state of aggregation as well as the concomitant metabolic changes of macrophages were investigated on LPS-activated RAW264.7 cells. Furthermore, we expressed PKM2 protein using *E. coli* system, and evaluated the effect of Shikonin on conformation of PKM2 protein by using size-exclusion chromatography. Finally, TEPP-46, an activator of PKM2 tetramerization, was used to validate the mechanism of Shikonin in DSS mice.

## Materials and methods

### Material and reagents

Shikonin (PubChem CID: 479503, HPLC≥98%) was purchased from DASF (Nanjing, China). Sulfasalazine was purchased from Fuda Pharmaceutical Co., Ltd (Shanghai, China). DSS (molecular weight of 36–50 kDa) was obtained from MP Biomedicals (Irvine, CA, United States). TEPP-46 (ML-265) were purchased from MedChemExpress (NJ, United States). Recombinant murine IFN-γ (315-05) were purchased from PeproTech (NJ, United States). LPS (L2880) and 4,6-Diamidino-2-phenylindole dihydrochloride (DAPI, D8417) were purchased from Sigma-Aldrich. Cell culture media, fetal bovine serum (FBS), and penicillin-streptomycin were obtained from Thermo Fisher Scientific (Waltham, MA, United States).

### Animal experiment

Male Balb/c mice (20–26 g) were purchased from Guangdong Medical Experimental Animal Center and housed in specific pathogen-free (SPF) conditions with constant temperature (20–25°C), humidity (65–70%) and a 12 h light/dark cycle. Experimental protocols were strictly following the ethical regulation of the Committee for Animal Care and Use at Guangzhou University of Chinese Medicine and the Guide for the Care and Use of Laboratory Animals. As previously described, mice in DSS groups received 3% DSS dissolved in drinking water for consecutive 7 days, while the control mice were given same volume of distilled water (Mai et al., 2019). Shikonin (6.125 mg/kg, 12.5 mg/kg, 25 mg/kg) and SASP (200 mg/kg) were gavaged once per day for seven consecutive days (Andujar et al., 2012; Han et al., 2021). Accordingly, TEPP-46 (10 mg/kg) was injected intraperitoneally for 7 days, where parallel groups received physiological saline.

### Cell culture

RAW264.7 cells were purchased from cell bank of Shanghai (Institute of Biochemistry and Cell Biology, Shanghai, China). The cells were grown in DMEM containing 10% FBS and 1% penicillin-streptomycin antibiotic at 37 °C and 5% CO_2_ in an incubator. Cells were pre-treated 1 h with Shikonin (0.5, 1, 2 μM), and then challenged with LPS (250 ng/ml) and IFN-γ (100 ng/ml) for 24 h to induce M1 type macrophage (Wu et al., 2021). Each experiment was repeated in three independent parallel tests.

### Hematoxylin-eosin staining

On the seventh day, all mice were euthanized for histologic analysis. The colon tissue was fixed in 4% paraformaldehyde, and paraffin-embedded samples were stained with H&E.

### ELISA

Colons kept at -80°C were homogenized. The supernatant was collected to measure the concentrations of IL-6, TNF-α and IL-10 using commercial ELISA kits following the manufacturer’s instructions (Invitrogen, Carlsbad, California, United States). The protein concentration was measured using a BCA protein assay kit (ComWin Biotech, Beijing, China) (Wu et al., 2021).

### Immunohistochemistry

The tissues were fixed with 4% paraformaldehyde, the tissue sections were deparaffinized with xylene, hydrated with gradient ethanol, and then incubated with anti-PKM2 (4053S, Cell Signaling Technology, United States) or F4/80 (70076S, Cell Signaling Technology, United States) or iNOS (ab3523, Abcam, United Kingdom) antibodies. After being developed by diaminobenzidine, the sections were observed using an optical microscope (Olympus BX51).

### Pyruvate and lactate and glucose consumption determination

The concentrations of pyruvate and lactate were measured using pyruvate kinase (PK) activity detection kit (Solarbio, China), lactate detection kit (Nanjing jiancheng, China) and glucose consumption (Nanjing jiancheng, China) according to manufacturer’s instructions.

### Real-time quantitative polymerase chain reaction and genetic identification

RNA was extracted using trizol (Invitrogen, United States) and first-strand cDNA synthesis was performed using PCR kit (TaKaRa, Japan). The mRNA levels of interleukin IL-1β, IL-6, IL-10 and TNF-α were assessed using quantitative PCR with Perfecta SYBR Green PCR kit (TaKaRa, Japan). *IL-1*β (5′-GAA​ATG​CCA​CCT​TTT​GAC​AGT​G-3′ and 5′-TGG​ATG​CTC​TCA​TCA​GGA​CAG-3′), *IL-6* (5′-TCT​ATA​CCA​CTT​CAC​AAG​TCG​GA-3′ and 5′-GAA​TTG​CCA​TTG​CAC​AAC​TCT​TT-3′), *IL-10* (5′-GGCGCTGTCATCGATTTCTC-3′and 5′-ATG​GCC​TTG​TAG​ACA​CCT​TGG-3′), *TNF-*α (5′-CAG​GCG​GTG​CCT​ATG​TCT​C-3′ and 5′-CGA​TCA​CCC​CGA​AGT​TCA​GTA​G-3′) and *GAPDH* (5′-TTG​ATG​GCA​ACA​ATC​TCC​AC-3′ and 5′-CGT​CCC​GTA​GAC​AAA​ATG​GT-3′), and relative expressions were calculated using the 2^−ΔΔCT^ method. Amplification reactions consisted of an initial denaturation at 95°C for 30 s, 40 cycles of denaturation at 95°C for 5 s, annealing at 60°C for 34 s, and extension at 95°C for 15 s.

### Western blot

Total protein was extracted using radio-immunoprecipitation assay (RIPA) (CW2333S, Kangweishiji, China) containing phosphatase inhibitor and a protease inhibitor, fractioned by SDS-PAGE and transferred onto a polyvinylidene difluoride (PVDF) membrane. PVDF membrane were incubated with anti-F4/80 antibody (70076S, Cell Signaling Technology, United States), anti-CD86 antibody (19589S, Cell Signaling Technology, United States), anti-IFN-γ antibody (ab133566, Abcam, United Kingdom), anti-iNOS antibody (ab3523, Abcam, United Kingdom), anti-PKM2 antibody (4053S, Cell Signaling Technology, United States), anti-p-PKM2 (8337S, Cell Signaling Technology, United States), and anti-Lamin B1(13435S, Cell Signaling Technology, United States) antibodies prior to probe with HRP conjugated secondary antibody (7074S, Cell Signaling Technology, United States). Immunoblots on the membrane were developed by the chemiluminescence (WBKL S0500, Millipore, United States) and photographed in an imaging station (Tanon-5200, Shanghai Tanon Technology, China).

### DSS crosslinking

Crosslinking was performed according to instructions of disuccinimidyl suberate (DSS) crosslinkers (Thermo Scientific) (Palsson-McDermott et al., 2015). Cells were pre-treated 1 h with Shikonin (0.5, 1, 2 μM), and then challenged with LPS (250 ng/ml) and IFN-γ (100 ng/ml) for 24 h, cells were collected and washed two times with ice-cold PBS. Add the DSS solution to a final concentration of 500 nМ, and incubate the reaction mixture for 30 min at room temperature. Add the Quench Solution to a final concentration of 1M Tris-Hcl (pH = 7.5) and incubate the quenching reaction for 15 min at room temperature. Lysates were analyzed by western blot.

### Construction of PKM2 plasmid

A gene encoding mouse PKM2 (AA 1–531) from mouse cDNA were subcloned into the pET28a (+). The nucleotide primers used for PCR were designed to consist of XhoI and NheI restriction sites flanking the gene. The following primers were used: forward, 5′- GCG​GCA​GCG​CTA​GCA​TGC​CGA​AGC CACACAGTGAA-3′; reverse, 5′- TTG​CAC​TTC​TCG​AGT​CAA​GGT​ACA​GGC​ACT ACACG-3′. The amplified gene inserts and the pET28a (+) vector were digested by XhoI and NheI. The insert was ligated into the vector at an insert-to-vector ratio of 3:1. The plasmids was evaluated by colony PCR and sequenced.

### PKM2 protein expression and purification

The recombinant plasmid was transformed into *E. coli* BL21 (DE3) cells for expression. The cells were grown in LB medium at 37°C until the optical density at 600 nm (OD_600_) reached 0.6-0.8, induced with 0.2 mM isopropyl-β-D-thiogalactoside (IPTG) at 25°C for 10 h. Cells were lysed in buffer including 40 mM Tris-HCl 8.0, 250 mM NaCl, 10 mM imidazole, 4 mM *β*-ME and 0.1 mM PMSF. Then PKM2 protein was the first purified by Ni-NTA affinity resin and eluted in buffer containing 250 mM imidazole, and purified again by HiTrap QHP 1 ml (GE, United States) and Superdex 200 increase 10/300 GL resin (GE, United States).

### Size-exclusion chromatography

Finally, the protein purified by HiTrap QHP 1 ml (GE, United States) incubated with or without Shikonin was separated by Superdex 200 increase 10/300 GL (GE Healthcare) to become homogeneous in solution. The column was first equilibrated with buffer (150 mM NaCl, 20 mM Tris-HCl), and the target protein was concentrated. The aliquoted protein was flash-frozen and stored at -80°Cuntil further use.

### Molecular docking

The 3D structure of PKM2 was obtained from PDB (PDB ID:1T5A). The 3D structure of Shikonin was obtained from Pubchem Compound. Schrodinger software was used to add hydrogens in the protein and ligand. Finally, the ligand was docked by the ligand docking. According to the principle, a high docking score with more energy released was considered to represent a better docking effect.

### Statistics

Data are presented as mean ± SD. The significance of the intergroup differences was analyzed with one-way analysis of variance (one-way-ANOVA) and Dunn´s multiple comparison tests using GraphPad Prism 8.0 software. A value of *p* < 0.05 was considered as significant difference.

## Results

### Shikonin alleviated the symptoms of DSS-induced mice colitis

**FIGURE 1:**
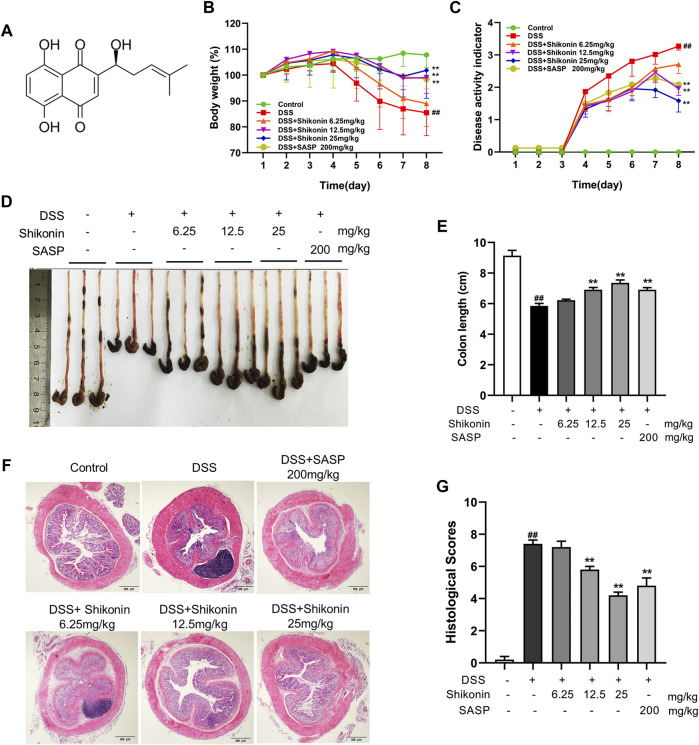
Shikonin attenuated the symptoms of DSS-induced mice colitis. Chemical structure of Shikonin **(A)**. The body weight **(B)** and disease activity index (DAI) of mice **(C)** during the course of colitis. Representative images **(D)** and statistics **(E)** of colon length in each group. Representative images **(F)** of H&E-stained colon tissue samples (scale bar, 500 μm) and histological scores **(G)** of colonic tissues. Data are shown as the mean ± SD, *n* = 5–8. ##*p* < 0.01, compared to the control group. **p < 0.01, compared to the DSS group.

To evaluate the protection of Shikonin on experimental colitis, a mice colitis model was established via 3% DSS solution for consecutive 7 days. Compared with animal in control group, mice in DSS group had significantly lower bodyweights (Figure 1B) and higher DAI scores (Figure 1C) calculated as the sum scores of weight loss, stool consistency and rectal bleeding. DSS mice also had shorter colons than control mice (Figures 1D,E). These data demonstrated that DSS induced colonic edema, gross bleeding and alterations in stool consistency. By contrast, mice in Shikonin (6.25, 12.5 and 25 mg/kg) and Sulfasalazine (as positive control) groups gradually restored their bodyweights and colon lengths, and their DAI scores decreased. In addition, histopathological analysis following H&E staining showed that DSS caused severe enteric mucosal injury including loss of epithelial crypts, disruption of mucosal barrier and infiltration of inflammatory cells, which directly leading to higher histological score (Figures 1F,G). However, all characteristic features were prevented by oral Shikonin supplementation. These results revealed that Shikonin alleviated the gross symptoms of DSS-induced mice colitis and alleviated colonic injury.

### Shikonin inhibited M1 macrophage polarization in DSS-mice

**FIGURE 2:**
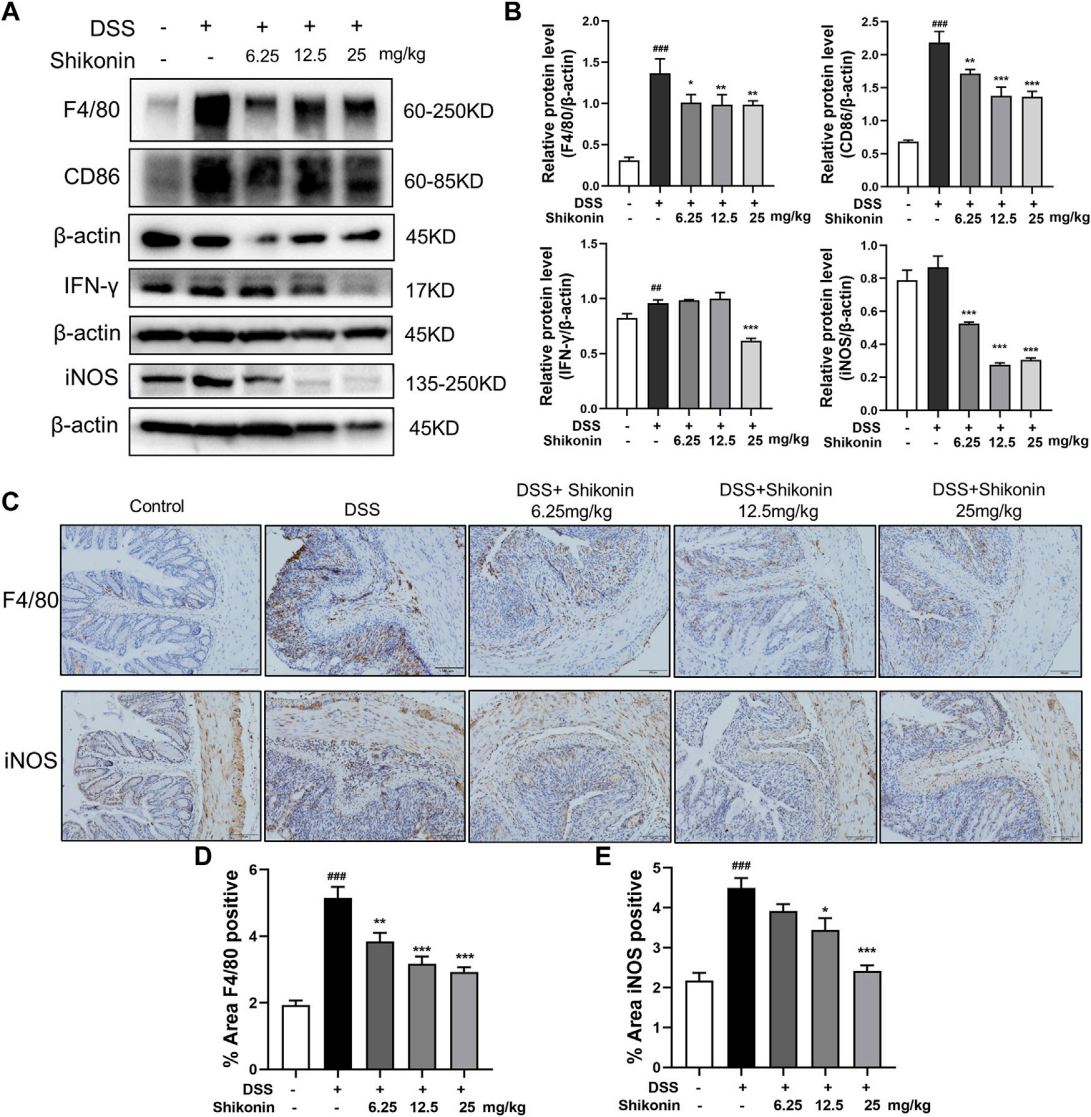
Shikonin inhibited macrophage polarization in DSS-mice. The protein level **(A)** and statistics **(B)** of F4/80, CD86, IFN-γ and iNOS measured by Western blots. The F4/80and iNOS **(C)** level of colitis were detected by immunohistochemistry assay with statistics **(D–E)**. Data are shown as the mean ± SD, *n* = 5–8. ##*p* < 0.01, ###*p* < 0.001, compared to the control group. *p < 0.05, **p < 0.01, ***p < 0.001, compared to the DSS group.

**FIGURE 3:**
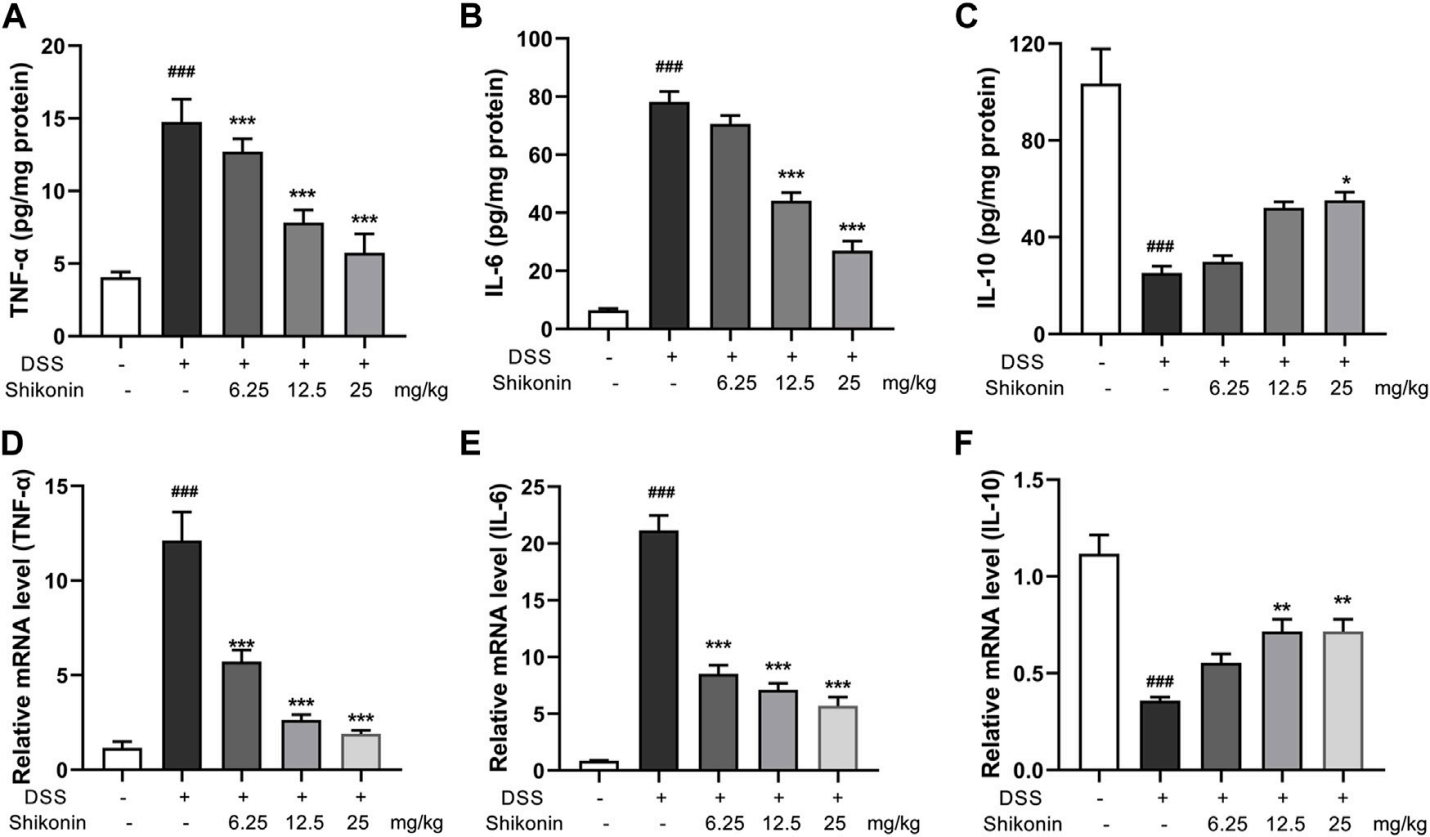
Effects of Shikonin on colon cytokines in DSS-induced mice colitis. Colon inflammatory factors, TNF-α **(A)**, IL-6 **(B)**, IL-10 **(C)** in the colon of acute colitis mice were determined by ELISA. Relative mRNA expression of TNF-α **(D)**, IL-6 **(E)**, IL-10 **(F)** in the colon were evaluated by RT-qPCR. Data are shown as the mean ± SD, *n* = 5–8. ###*p* < 0.001, compared to the control group. *p < 0.05, **p < 0.01, ***p < 0.001, compared to the DSS group.

Macrophage infiltration and secretion of inflammatory factors is recognized as evaluative parameters for colitis, and M1 phenotype of macrophage is widely believed to be proinflammatory (Na et al., 2019). As shown in Figures 2A,B, Shikonin (6.25, 12.5 and 25 mg/kg) evidently suppressed both F4/80 which is the maker of activated macrophage and M1 macrophage markers including CD86, IFN-γ and iNOS in DSS-mice. Furthermore, F4/80 and iNOS detected by IHC were fulfilled similar results which furthermore suggested Shikonin suppressed M1 macrophage polarization in the colonic lamina propria of colitis mice (Figure 2C, E). In addition, Shikonin treatment significantly reduced the level of inflammatory factors such as IL-6 and TNF-a, while elevated IL-10 level (Figures 3A–F). These findings indicated that Shikonin inhibited M1 macrophage polarization and infiltration in DSS-mice, and ameliorated colonic inflammation.

### Shikonin inhibited the aerobic glycolysis and PKM2 in M1 macrophages

**FIGURE 4:**
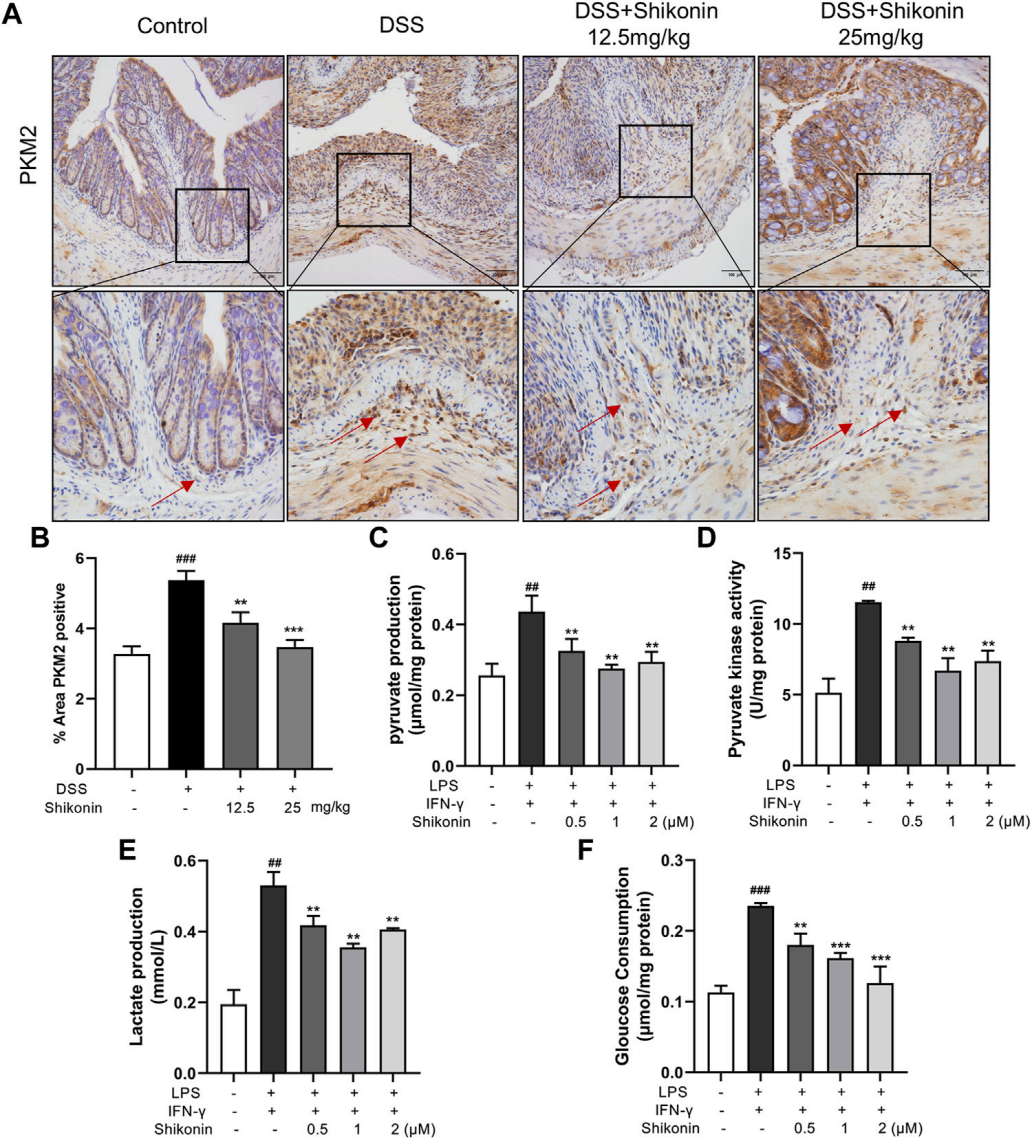
Shikonin inhibited PKM2 *in vivo* and its enzymatic pyruvate kinase activity and lactate production in RAW264.7 cells. Expression of PKM2 in colon of colitis mice was detected by immunohistochemistry assay **(A)** with statistics **(B)**. Pyruvate production **(C)**, pyruvate kinase activity **(D)**, lactate production **(E)** and gloucose consumption **(F)** were measured in Raw264.7 cells. Data are shown as the mean ± SD, n = 6 in Figure 4A, and *n* = 3 in Figures 4B–D. ##*p* < 0.01, compared to the control group. **p < 0.01, compared to the LPS + IFN-γ group.

**FIGURE 5:**
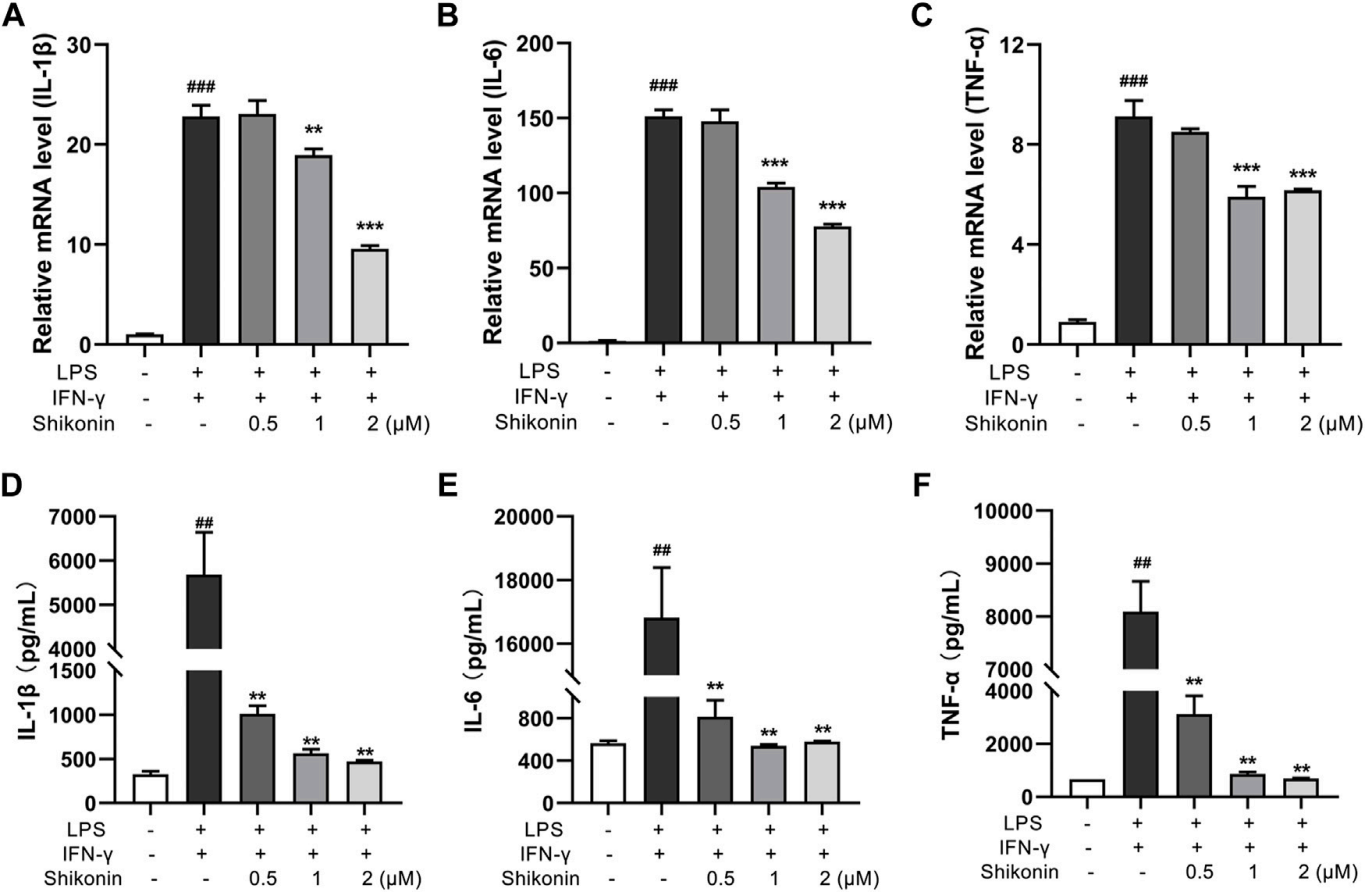
Shikonin inhibited proinflammatory cytokines in RAW264.7 cells. Relative mRNA expression of IL-1β **(A)**, IL-6 **(B)**, TNF-α **(C)** in RAW264.7 cells were evaluated by RT-qPCR. The levels of IL-1β **(D)**, IL-6 **(E)** and TNF-α **(F)** in RAW264.7 cells were determined by ELISA. Data are shown as the mean ± SD, *n* = 3. ##*p* < 0.01, ###*p* < 0.001, compared to the control group. **p < 0.01, ***p < 0.001, compared to the LPS + IFN-γ group.

Aerobic glycolysis is a crucial metabolic event for M1 macrophages accompanied by increased lactate production. PKM2 is one of the rate-limiting enzymes in glycolysis, which can rapidly regulate its metabolic activity by changing its distribution in cytoplasm and nucleus (Liu et al., 2021). Accordingly, PKM2 in the colonic lamina propria of colitis mice were reduced after treatment with Shikonin (12.5 and 25 mg/kg) compared with DSS group (Figures 4A,B). Next, pyruvate and pyruvate kinase activity were detected in RAW264.7 cells. As shown in Figures 4C,D, compared with LPS + IFN-γ group, Shikonin dose-dependently suppressed both pyruvate level and pyruvate kinase activity. In addition, Shikonin (0.5, 1.0 and 2.0 μM) significantly inhibited lactate production and gloucose consumption in LPS + IFN-γ simulated RAW264.7 cells (Figures 4E,F). Furthermore, both mRNA (Figures 5A–C) and protein expression (Figures 5D–F) of IL-1β, IL-6 and TNF-α were decreased in LPS + IFN-γ simulated RAW264.7 cells, which was consistent with the results in animals. These data demonstrated that Shikonin suppressed aerobic glycolysis of macrophage, which may be mediated by inhibiting PKM2 expression.

### Shikonin inhibited PKM2 nuclear translocation and the formation of both PKM2 dimer and tetramer

**FIGURE 6:**
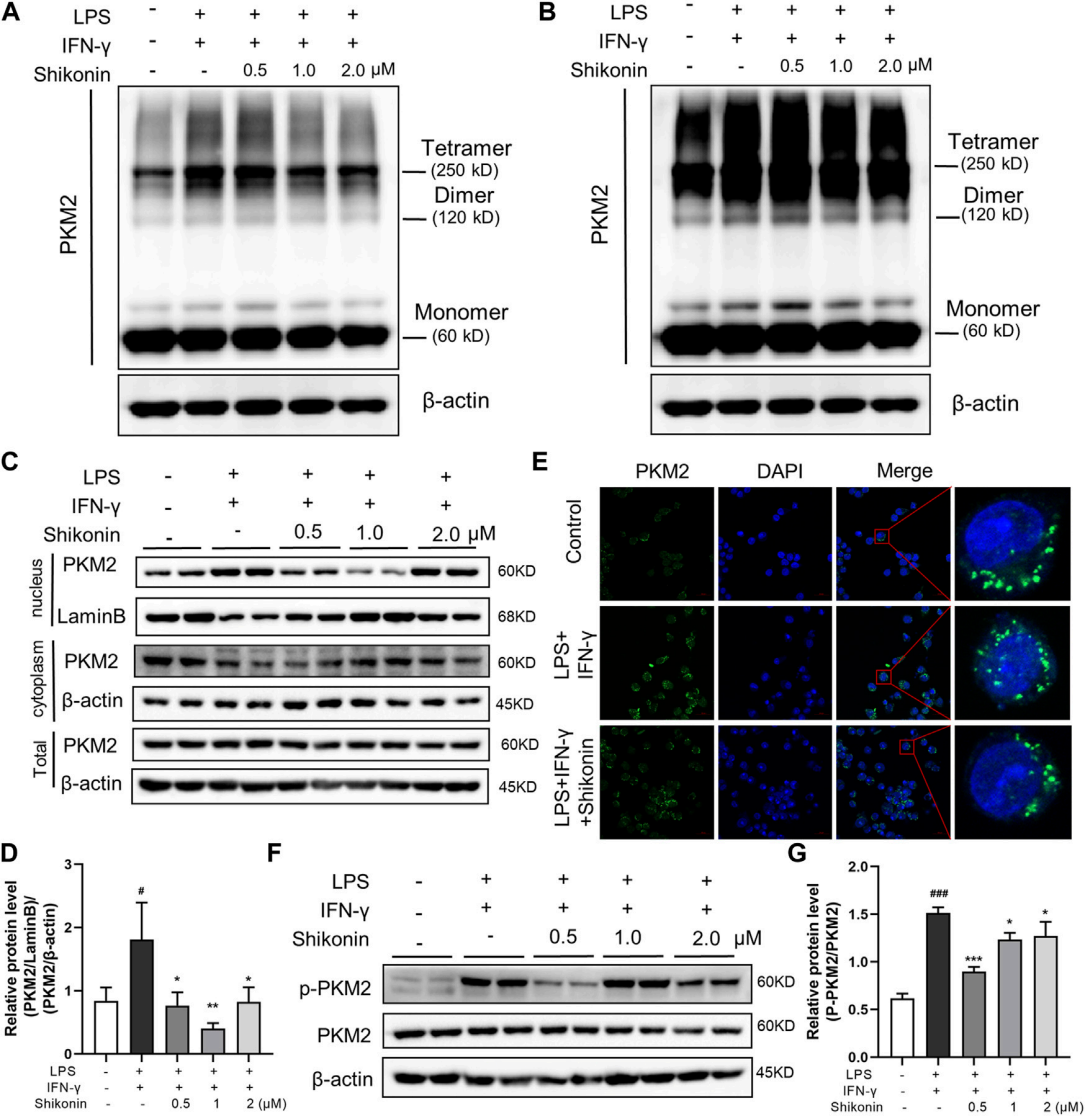
Shikonin regulated PKM2 polymerization and inhibited its nuclear translocation and phosphorylation and in RAW264.7 cells. Cells were collected and cross-linked with DSS. Tetramer, dimer and monomer form of PKM2 in RAW264.7 cells were analyzed by western blot **(A,B)**. **(A)** showed the effect of short exposure and **(B)** showed the effect of long exposure. PKM2 in nucleus and in cytoplasm and the statistics were detected by western blots **(C)** with statistics **(D)**. Cellular localization of PKM2 was analyzed by immunofluorescence staining **(E)**. The nucleus was stained with DAPI. Expression of P-PKM2 and PKM2 and were detected by western blots **(F)** with the statistics **(G)**. Data are shown as the mean ± SD, *n* = 3. #*p* < 0.05, ###*p* < 0.001, compared to the control group. *p < 0.05, **p < 0.01, ***p < 0.001, compared to the LPS + IFN-γ group.

PKM2 has three forms of allosteric structure: monomer, dimer and tetramer, with different cellular location and diverse functions in glycolysis and OXPHOS, respectively. PKM2 in the dimer state can enter the nuclear to regulate gene expression of pro-inflammatory factors (Zhang et al., 2019). Next, we explored the specific configuration of PKM2 inhibited by Shikonin, and determined PKM2 protein expression in nucleus. DSS cross-linking was carried out to stabilize the allosteric structure of PKM2, and the blots revealed that Shikonin (0.5, 1.0 and 2.0 μM) dose-dependently inhibited both PKM2 tetramer and dimer in LPS + IFN-γ induced RAW264.7 cells, while PKM2 monomer showed no obvious changes (Figures 6A,B). Furthermore, it was also clearly showed that Shikonin prevented PKM2 transferring from cytoplasm to nucleus as shown in Figures 6C–E. As shown in Figure 6C, the total PKM2 expression showed no differences among Control, Model (LPS + IFN-γ) and Shikonin groups. Whereas, the PKM2 expression in nucleus elevated when it is calculated as the ratio of PKM2/LaminB over cytoplasm PKM2/β-actin. In addition, PKM2 phosphorylation at different sites shows diverse function and different metabolic pathway. Y105 phosphorylation is indicator of dimer formation, as it prevents PKM2 tetramer configuration, further promoting the Warburg effect (Hitosugi et al., 2009). Current data showed that Shikonin blocked Y105 phosphorylation of PKM2 (Figures 6F,G). All of these findings showed that Shikonin suppressed the nuclear translocation of PKM2 dimer and reduced PKM2 tetramerization.

### Shikonin modulated the aggregation state of PKM2

**FIGURE 7:**
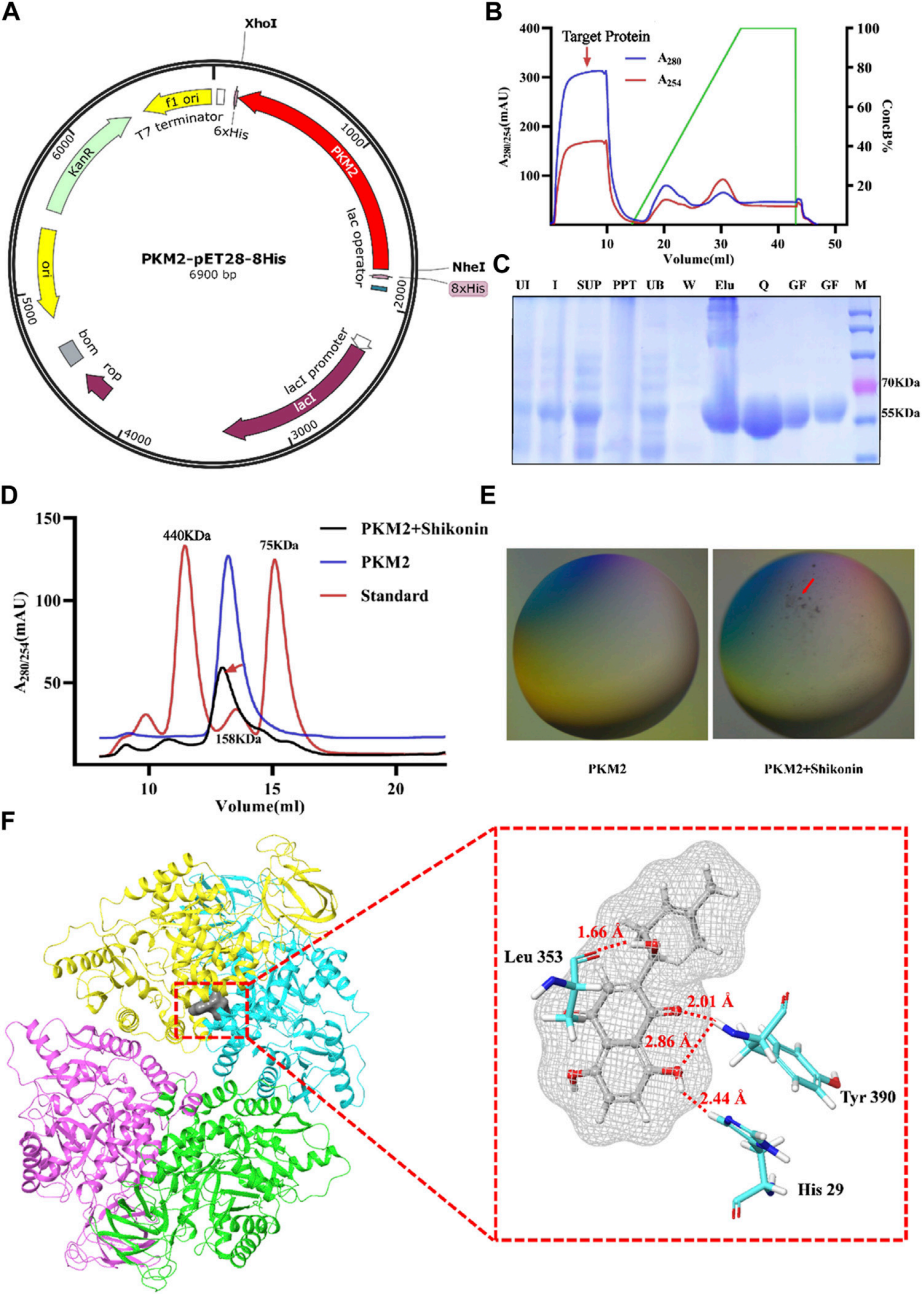
Shikonin bound to PKM2 to promote the polymerization. Map of the PKM2-pET28a (+) plasmid **(A).** Chromatogram of protein purification with Hi trap QHP **(B)**. The expression and purification of PKM2, and the purity was analyzed by 12% SDS-PAGE gels. Lane 1, uninitiated; Lane 2, initiated; Lane 3, supernatant fraction; Lane 4, precipitation fraction; Lane 5, fraction not bound to Ni-NTA; Lane 6, fraction eluted with buffer A; Lane 7, collected by buffer B; Lane 8, collected by Hi trap QHP; Lane 9-10, purified protein by Superdex 200 increase 10/300 GL without or with Shikonin **(C)**. Chromatogram of protein purification with Superdex 200 increase 10/300 GL without or with Shikonin **(D)**. Two images under a polarizing microscope **(E)**. Structure view of PKM2 and Shikonin, and gray color represents Shikonin. The picture shows the amino acid residues with hydrogen bonds as red lines **(F)**.

In order to further identify the mechanism of Shikonin and PKM2, we inserted the PKM2 gene into pET28a (+) vector for overexpression and the red represents PKM2 gene *in vitro* (Figure 7A). After the first purification step using Ni-NTA agarose affinity chromatograph, the PKM2 protein (59 KDa) was separated from the supernatant of whole cell lysate with a high purity of ∼70% (Figure 7C). An anion exchange chromatography QHP column was used to obtain the pure protein. As is shown in Figure 7B, the unbound fraction containing target protein was collected and the purity was verified by 12% SDS-PAGE (Figure 7C). In order to explore the effect of Shikonin on PKM2 protein aggregation, we evaluated the molecular weight of PKM2 protein by using size exclusion chromatography. PKM2 existed as a mixture of dimer and tetramer in solution, which average molecular weight was around 150 kDa and the mixture was eluted out of the chromatographic column at the volume of 13 ml. In the presence of Shikonin, two eluting peaks representing macropolymers of PKM2 were increased at the elution volume of 7 and 12 ml respectively. However, the dimer and tetramer mixture were decreased and left-shift around the elution volume of 13 ml (Figure 7D). Each fraction was pooled and concentrated for the subsequent experiments. We screened the crystals, and under the polarizing microscope, we found that the protein which had been added with Shikonin, formed microscopic precipitations (Figure 7E). To reveal the interaction mode of PKM2 and Shikonin, we used molecular docking. The docking score ranged from -6.563 to -7.900. The binding structure with the highest score and the highest energy was released to analyze. The residues His 29, Leu 353 and Tyr 390 of PKM2 formed hydrogen bonds with hydroxyl groups with Shikonin, respectively. (Figure 7F). All these data reflected that Shikonin bound to PKM2 and modulated the protein aggregation form of PKM2 into polymer.

### Shikonin ameliorated mice colitis via modulating PKM2 into polymer instead of promoting PKM2 tetramer

**FIGURE 8:**
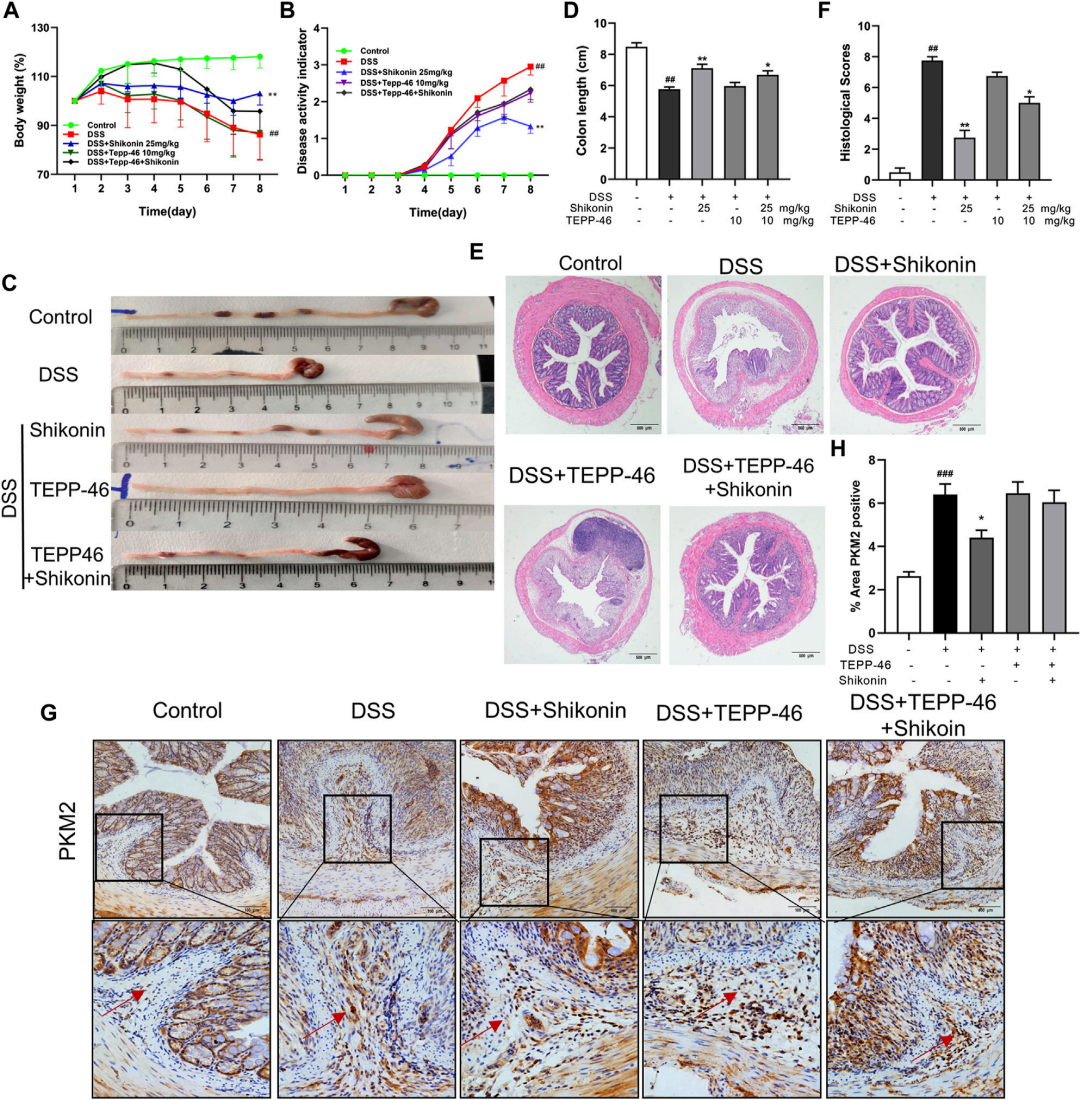
Shikonin enhanced the anti-UC effect of TEPP-46 in colitis mice. The body weight **(A)** and DAI **(B)** of mice during the course of colitis. Representative images **(C)** and statistics **(D)** of colon length in each group. Representative images **(E)** of H&E-stained colon tissue samples (scale bar, 500 μm) and histological scores **(F)** of colonic tissues. Expression of PKM2 in colon of colitis mice were detected by immunohistochemistry assay **(G)** with the statistics **(H)**. Data are shown as the mean ± SD, *n* = 5–8. ##*p* < 0.01, compared to the control group. *p < 0.05, **p < 0.01, compared to the DSS group.

In order to validate whether Shikonin attenuated colitis via promoting PKM2 polymerization instead of tetramerization, TEPP-46, an activator of PKM2 tetramerization was used alone or in combination with Shikonin in DSS-mice. Compared with the parameters of DSS group, neither the body weights nor the DAI scores of TEPP-46 group have changed significantly (Figures 8A,B). Similarly, TEPP-46 did not remarkably improve the length and the injury of colon in DSS mice (Figures 8C–F). In contrast, when mice were treated with Shikonin, all of these disease features were improved effectively compared with DSS group (Figures 8A–F). Nevertheless, TEPP-46 intervention obviously blocked the ameliorative effect of Shikonin. In addition, PKM2 positive expression in lamina propria of Shikonin group was reduced in comparison to DSS group, whereas the TEPP-46 and TEPP46 + Shikonin groups did not show evident changes (Figures 8G,H).

## Discussion

Recent progresses in immunometabolism is revolutionizing the treatment of diseases including UC, in which dysregulated immune responses have an important role (Kaluzna et al., 2022). Macrophages are crucial immune cells in colon mucosa. Although intestinal macrophages play a vital role in protecting gut against exotic invasion and damage, classically activated M1 macrophages, under the challenge of intestinal microbes and contents, undergo a metabolic shift from resting state to a highly active aerobic glycolytic state. Such reprogramming may cause macrophages to be hyperresponsive, resulting in a dysregulated immune response in UC.

Macrophages consist of a heterogeneous population and play several diverse functions in homeostatic and immune responses. Therefore, crucial events in macrophage functional and metabolic programme are potential therapeutic targets to relieve inflammation. Here we found that Shikonin inhibited M1 activation of macrophages and decreased PKM2 expression in macrophages distributed in lamina propria of mice colon. Furthermore, a modulation on PKM2 polymerization was identified as the effective target of Shikonin.

Firstly, Shikonin remarkably ameliorated the symptoms of colitis in DSS mice. As known that DAI, bodyweight loss, colonic length and histological scores are main parameters used to evaluate the severity of UC(Goyal et al., 2014). Shikonin treatments prevented loss of bodyweights, extended colon lengths, inhibited inflammatory infiltration, suppressed inflammatory cytokines involving IL-6, TNF-α, IFN-γ and iNOS, and elevated colonic IL-10. These data are consistent with ameliorating effects of Shikonin on mice colitis reported previously (Han et al., 2021). In addition, immunohistochemistry and western blot results showed that Shikonin inhibited M1 macrophage polarization in colonic lamina of UC mice. All these suggest that Shikonin protect mice from DSS induced colitis by suppressing M1 polarization of macrophages and mitigating inflammatory responses.

The striking feature of M1 macrophage is metabolic reprogramming of aerobic glycolysis, also known as Warburg effect, in which glucose metabolites pathway is switched from entering tricarboxylic acid cycle into the pentose phosphate pathway, resulting in accumulation of pyruvate and lactate, as well as transcription of pro-inflammatory cytokines. *In vitro* study on RAW264.7 cells showed that LPS + IFN-γ induced M1 polarization of macrophages together with accumulation of pyruvate and lactate. In contrast, Shikonin decreased proinflammatory cytokines in M1 macrophages and attenuated the accumulation of metabolites, indicating that Shikonin reversed glycolytic reprogramming in macrophages.

PKM2 is currently considered to be the interception of glucose metabolism. Elevated PKM2 expression in M1 macrophages enables the metabolic switch from oxidative phosphorylation to aerobic glycolysis (Luo et al., 2011). PKM2 exists mainly as either dimeric form with low-pyruvate kinase activity or tetrameric form with high-pyruvate kinase activity. Although both forms of PKM2 are consist of same inactive monomer, different states of aggregation actually mediate different functions in glucose metabolism. PKM2 tetramer has a higher affinity with its substrate phosphoenolpyruvate and catalyzes the production of pyruvate. Nevertheless, PKM2 dimer mainly converts pyruvate from entering TCA cycle into the anabolic pathway, accumulates the upstream products of glycolysis and allows them to synthesize to biological macromolecules needed for cell growth and proliferation. Shikonin is a recognized inhibitor of PKM2, however, it remains elusive which conformation of PKM2 is inhibited by PKM2. Previous data reports that M1 macrophages predominantly express dimeric form of PKM2, whereas normal proliferating cells express the high-activity tetrameric form (Liu et al., 2021). In this study, a cross-linking method was used to simultaneously determine the dimeric and tetrameric forms of PKM2 protein in macrophages. The result showed that LPS + IFN-γ challenged macrophage to M1 polarization and expressed high levels of both PKM2 dimer and tetramer, indicating activation on both glycolysis and anabolic pathways. However, Shikonin treatment decreased both dimer and tetramer expression of PKM2, without influencing PKM2 monomer. These data suggest that Shikonin inhibited both pyruvate kinase and protein kinase of PKM2, which may collectively restrain metabolic reprogramming of M1 macrophages.

The allosteric formation of PKM2 is regulated by post-translational modifications. Phosphorylation at tyrosine 105 (Tyr 105) of PKM2 disrupts the tetrameric form while promote dimeric form, leading to a suppressed pyruvate kinase activity (Hitosugi et al., 2009). Furthermore, PKM2 contributes to the Warburg effect by translocating to nuclear and phosphorylating STAT3 or interacting with HIF-1α to regulate the expression of glycolytic genes and proinflammatory cytokines in macrophages (Palsson-McDermott et al., 2015; Shirai et al., 2016). Accordingly, current western blot results revealed that M1 macrophages challenged by LPS + IFN-γ expressed high levels of Tyr 105 phosphorylation and nuclear translocation of PKM2, indicating an enhanced glycolytic programme in activated macrophages. In contrast, Shikonin inhibited both Tyr 105 phosphorylation and nuclear translocation of PKM2 in RAW264.7 cells. These data further consolidated the inhibitory effect of Shikonin on both dimerization and tetramerization of PKM2.

To clarify how PKM2 influence the conformation of PKM2, we expressed PKM2 protein and examined whether there is a combination between Shikonin and PKM2. PKM2 gene were inserted into pET28a (+) vector and expressed on *E. coli*. PKM2 protein were purified *in vitro* by using agarose affinity chromatograph and anion exchange chromatography. Shikonin was directly added in a system containing PKM2 protein. We found that Shikonin decreased the formation of both dimer and tetramer, while promoted PKM2 to aggregate into macromolecular polymer. Consistently, under polarizing microscope, PKM2 protein formed microscopic precipitations after Shikonin treatment. The docking assay suggested that Shikonin formed hydrogen bonds with hydroxyl groups in the residues of His 29, Leu 353 and Tyr 390 of PKM2 protein. All these data demonstrated that Shikonin may modulate allosteric structure of PKM2 protein via forming hydrogen bonds and promoting aggregation of macropolymers. However, the specific interaction mechanism of Shikonin on PKM2 still merits further clarification.

Finally, TEPP-46, an activator of PKM2 tetramerization, was used to validate the mechanism of Shikonin on DSS mice. The results showed that activation of PKM2 tetramerization by TEPP-46 did not remarkably improve the length and the injury of colon in DSS mice. But TEPP-46 intervention obviously attenuated the ameliorative effect of Shikonin. Parallelly, TEPP46 antagonized the inhibitory effect of Shikonin on PKM2 positive expression in lamina propria of DSS mice. Collectively, these data suggest that promoting PKM2 pyruvate kinase activity via forming PKM2 tetramer by TEPP-46 may not significantly improve colitis, whereas a simultaneous inhibition on both dimerization and tetramerization by Shikonin significantly improved colitis.

In summary, Shikonin significantly ameliorated DSS-induced colitis, prevented intestinal injury and M1 macrophage infiltration in lamina propria of mice colon. Moreover, Shikonin inhibited both PKM2 dimerization and tetramerization, while promoted PKM2 to aggregate into macropolymer. These findings consolidated the therapeutic effect of Shikonin on DSS mice, and preliminarily elucidated that the immunoregulatory activity of Shikonin is possibly mediated by a metabolic regulatory on macrophages.

## Data Availability

The datasets presented in this study can be found in online repositories. The names of the repository/repositories and accession number(s) can be found in the article/Supplementary Material.
