# Supplementary material for: Shikonin ameliorated mice colitis by inhibiting dimerization and tetramerization of PKM2 in macrophages
Source: Front Pharmacol. 2022 Aug 17;13:926945. doi: 10.3389/fphar.2022.926945 (PMC9428403; doi:10.3389/fphar.2022.926945)
Supplement: Supplementary file 1 [file DataSheet2.docx]

Supplementary Figure 1. The purity of shikonin was analyzed by HPLC chromatograph with a UV detector at the wavelength of 516nm.


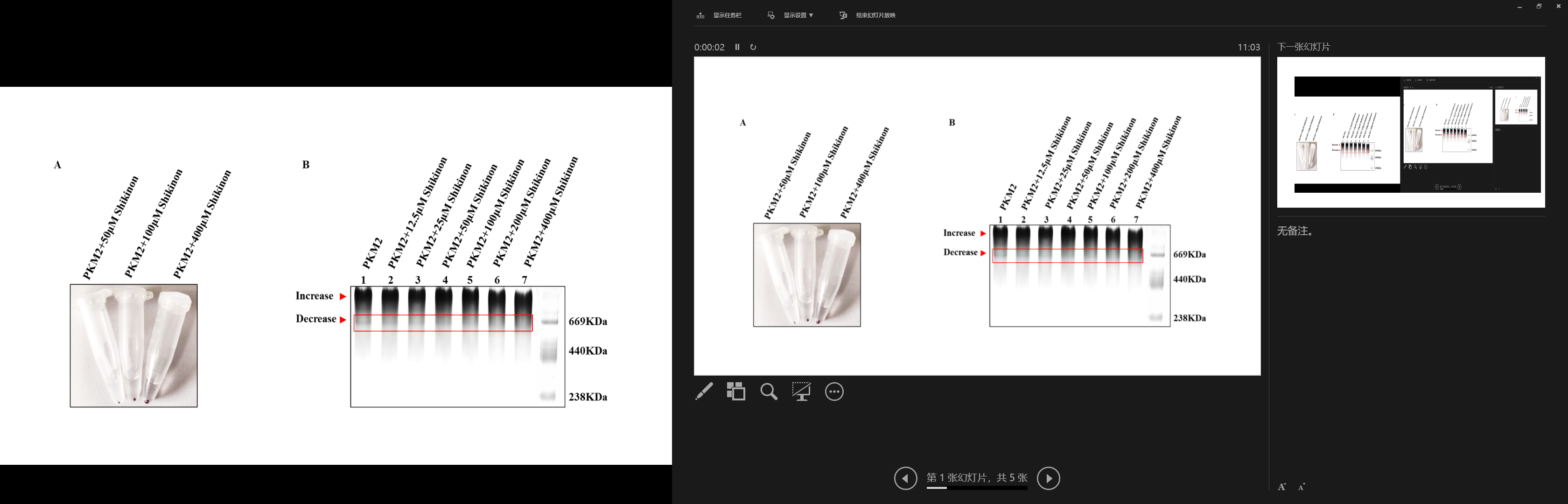


Supplementary Figure 2. Native-PAGE gel diagram analysis of PKM2. (A) Visible precipitation of PKM2 with different concentrations of Shikonin. (B) The native gel was stained with Coomassie brilliant blue and then decolorized. Lane 1, PKM2 protein only; Lane 2, PKM2 with 12.5 μM Shikonin; Lane 3, PKM2 with 25 μM Shikonin; Lane 4, PKM2 with 50 μM Shikonin; Lane 5, PKM2 with 100 μM Shikonin; Lane 6, PKM2 with 200 μM Shikonin; Lane 7, PKM2 with 400 μM Shikonin.
