# Supplementary material for: Shikonin ameliorated mice colitis by inhibiting dimerization and tetramerization of PKM2 in macrophages
Source: Front Pharmacol. 2022 Aug 17;13:926945. doi: 10.3389/fphar.2022.926945 (PMC9428403; doi:10.3389/fphar.2022.926945)
Supplement: Supplementary file 2 [file DataSheet1.docx]

Supplementary Material


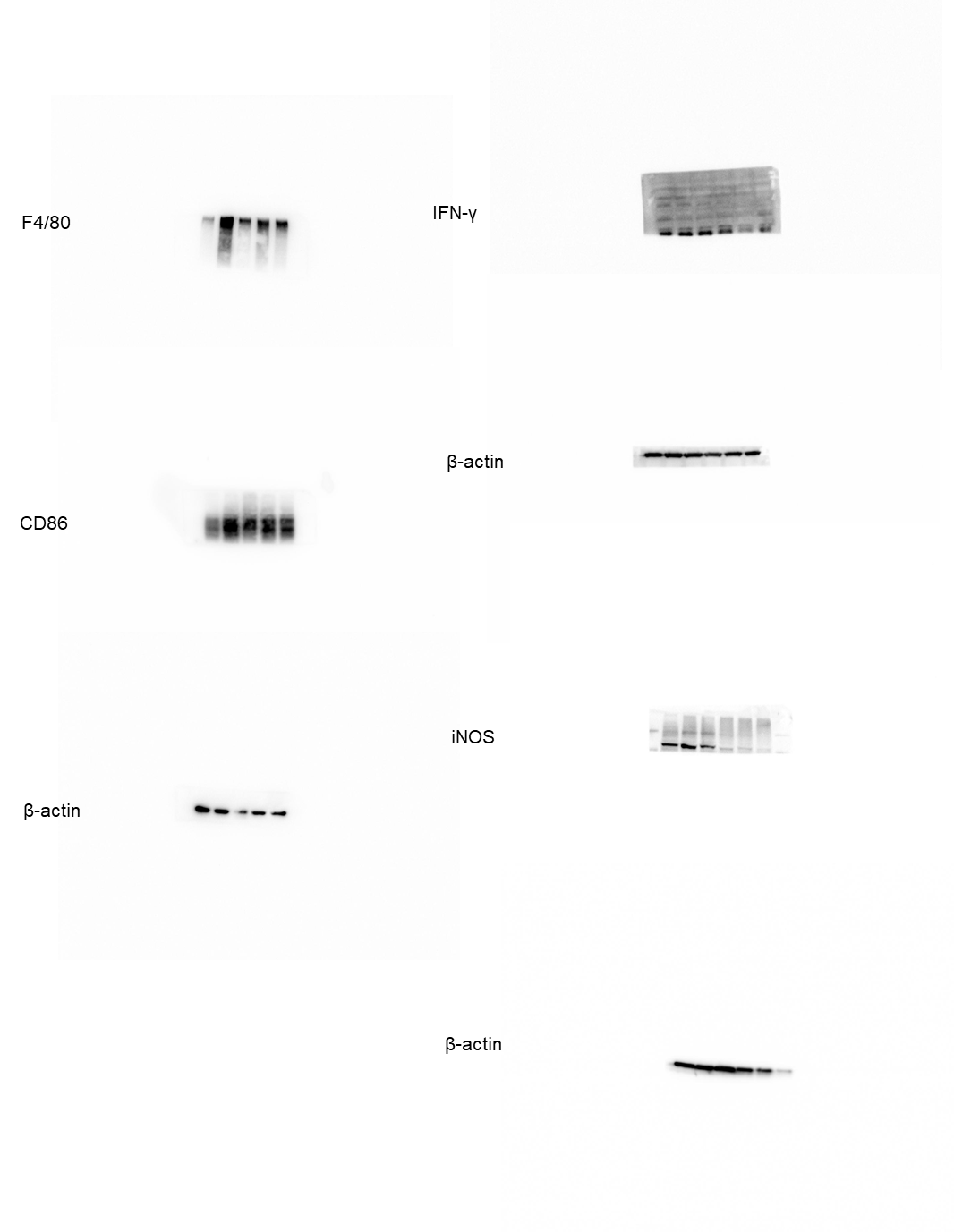


**Supplementary Figure 1.** The original data of Figure 2A of Manuscript


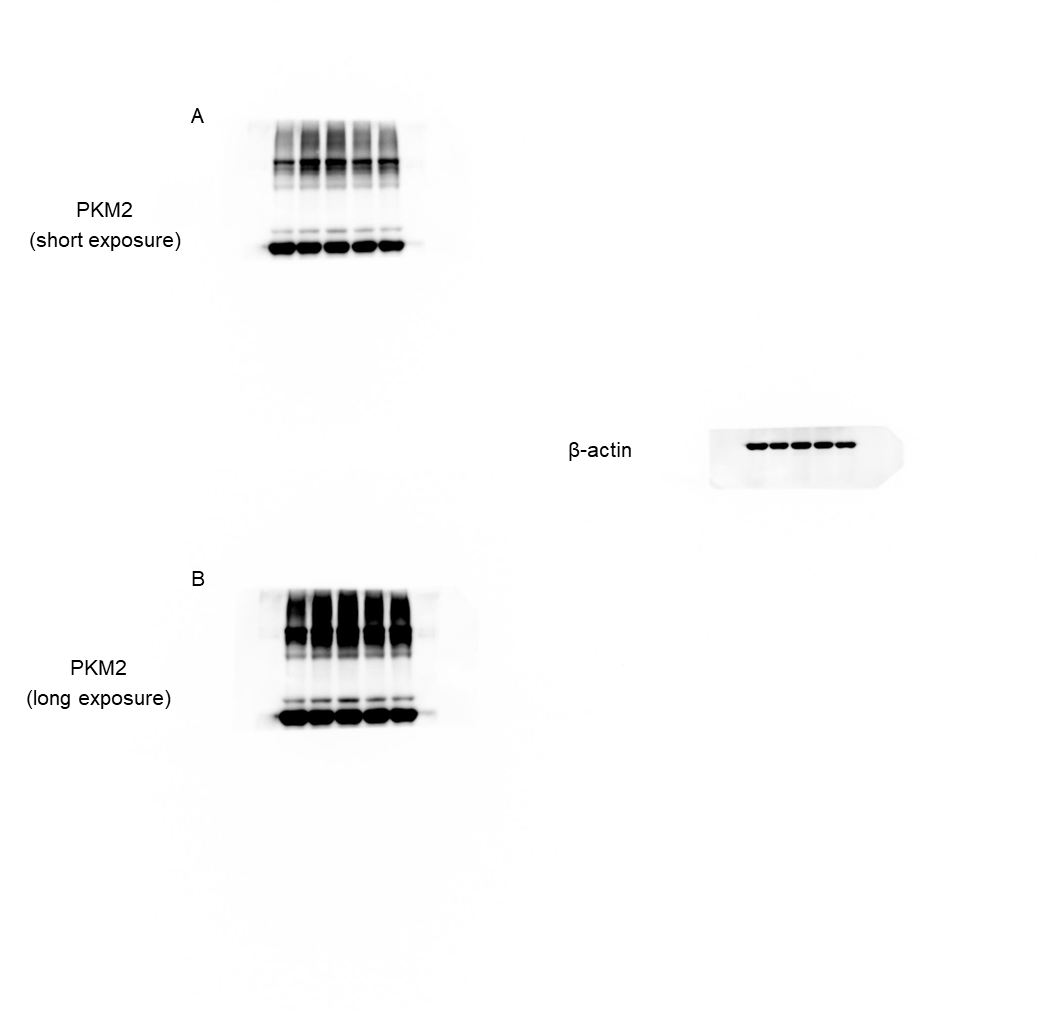


**Supplementary Figure 2.** The original data of Figures 6A and 6B of Manuscript


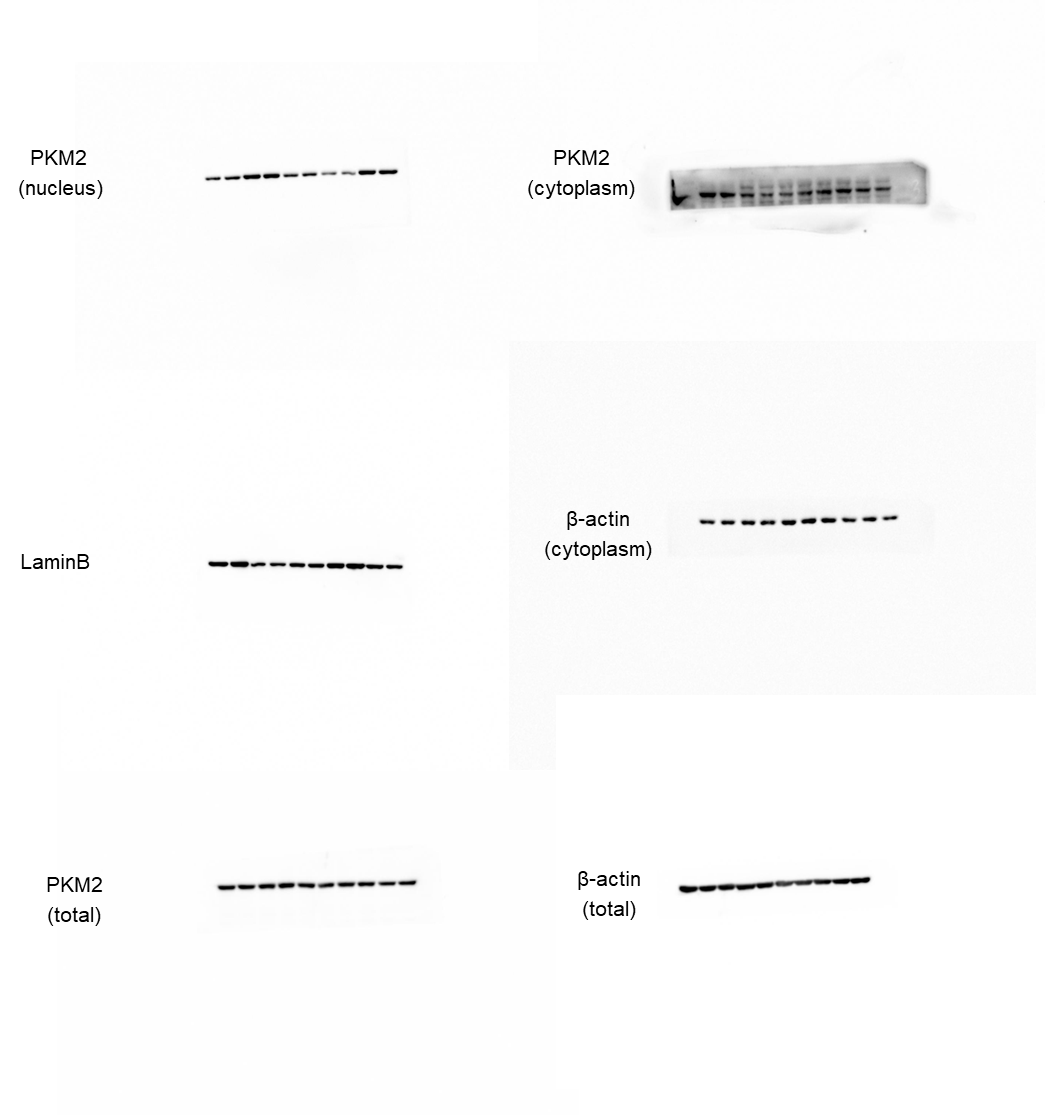


**Supplementary Figure 3.** The original data of Figure 6C of Manuscript


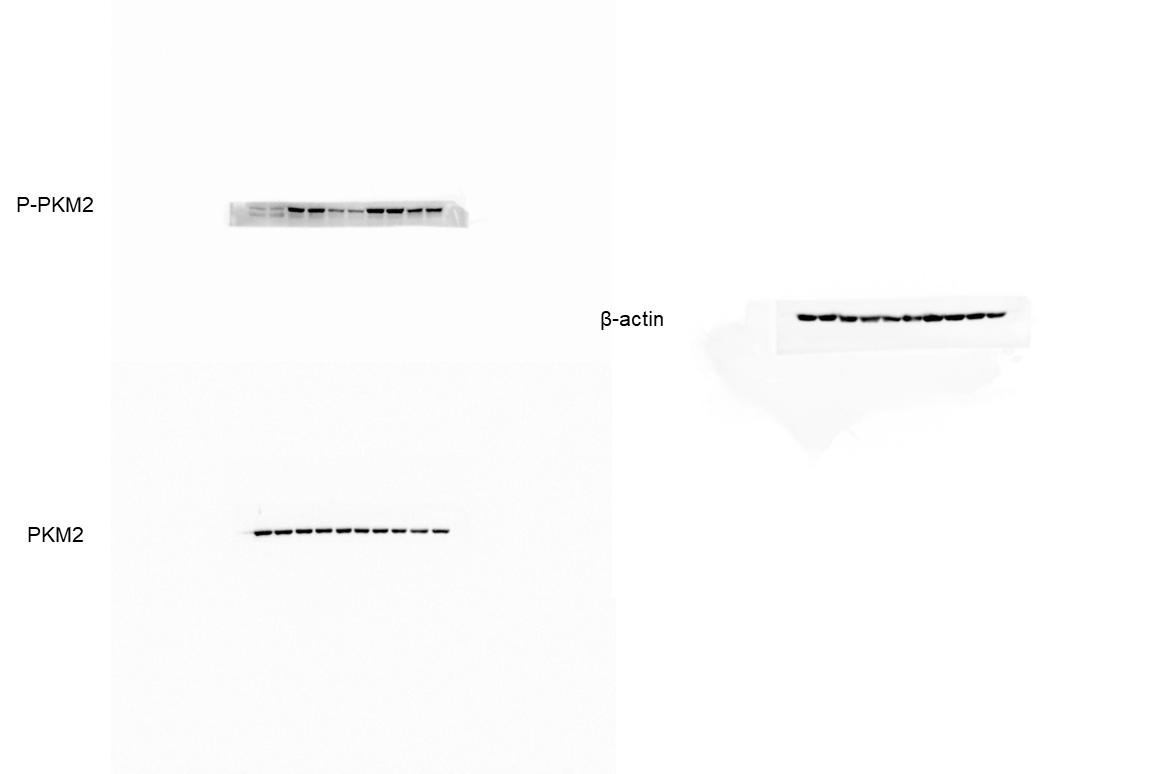


**Supplementary Figure 4.** The original data of Figure 6F of Manuscript
